# Understanding recruitment challenges in Swiss oncology trials: Patient voices from focus groups

**DOI:** 10.1016/j.conctc.2026.101661

**Published:** 2026-06-22

**Authors:** Sandro Tiziano Stoffel, Helene Seaward, Aksha Balakrishnan

**Affiliations:** a Institute of Pharmaceutical Medicine (ECPM), University of Basel, Basel, Switzerland; b Research Department of Behavioural Science and Health, University College London (UCL), London, UK; c Institute for Biomedical Ethics, University of Basel, Basel, Switzerland; d Department of Pharmaceutical Sciences, University of Basel, Basel, Switzerland

## 1 Introduction

Cancer remains a leading cause of morbidity and mortality worldwide, including in Switzerland, where it is the second most common cause of death, underscoring the critical need for ongoing oncology research and innovation in treatment strategies [1,2]. Clinical trials are essential for the development and evaluation of new cancer therapies, yet patient recruitment remains a persistent challenge, with a significant proportion of trials discontinued prematurely due to insufficient enrolment, resulting in wasted resources and unresolved research questions [3,4]. In Switzerland, it is estimated that approximately 25% of randomized controlled trials are prematurely discontinued [5,6].

Several studies have identified a complex array of barriers and facilitators influencing patients' decisions to participate in cancer clinical trials. Commonly reported barriers include fear of randomization, concerns about side effects, logistical challenges such as travel and time commitment, financial burden, lack of awareness, and distrust in the healthcare system [3,[7], [8], [9], [10], [11], [12]]. Facilitators often cited are hope for therapeutic benefit, trust in physicians, altruistic motivation, and the desire to contribute to scientific progress [[12], [13], [14], [15]]. The decision-making process is further shaped by patients’ emotional responses, personal values, family influence, and the quality of communication with healthcare professionals [[16], [17], [18], [19]]. Barriers and facilitators also depend on the disease stage, cancer type and cultural setting [4,11]. So far, no studies have investigated the lived experiences and perspectives of Swiss cancer patients. Moreover, recent qualitative syntheses emphasize the need for in-depth exploration of patient reasoning, values, and meaning-making processes, rather than solely generating insights for subsequent quantitative research [14,20,21].

### 1.1 The current study

This study aims to provide an in-depth, qualitative understanding of how Swiss cancer patients perceive and navigate the decision to participate in clinical trials. Using focus group interviews, the research seeks to capture the nuanced, lived experiences and meaning-making processes underlying patients’ choices, with the goal of informing more patient-centred recruitment strategies and enhancing ethical, effective communication in the Swiss context. The study is reported in accordance with the COREQ (Consolidated Criteria for Reporting Qualitative Research) guidelines [22].

## 2 Methods

### 2.1 Study design

This study employed an exploratory, inductive qualitative design using focus group interviews to gain a contextualised understanding of Swiss cancer patients' decision-making regarding clinical trial participation. Grounded in a constructivist paradigm, the approach aimed to elicit participants’ lived experiences, values, and reasoning processes [20,23].

### 2.2 Participants

Participants were recruited through purposive and snowball sampling in collaboration with patient advisory boards, cancer institutes, and regional cancer leagues in German-speaking Switzerland. Recruitment materials were distributed both digitally and in print, and presentations at oncology conferences supported outreach (see flyer in Fig. S1 and invitation email in Fig. S2 in the supplementary file). Eligibility criteria included being aged 18 or older, residing in Switzerland, having a confirmed cancer diagnosis, and possessing sufficient German language proficiency to participate in group discussions. We had originally aimed to recruit up to 15 participants; however, recruitment challenges resulted in a final sample of nine.

#### 2.2.1 Research team

Focus groups were facilitated by a team of two behavioural scientists with expertise in qualitative research and decision-making. The sessions were moderated by a trained qualitative researcher with support from a note taker. Participants were informed of the researchers’ backgrounds, roles, and gender prior to the sessions. No prior relationships existed between researchers and participants.

#### 2.2.2 Procedure

Three focus groups were conducted between December 2024 and January 2025 in Bern, Basel, and St. Gallen. Nine individuals participated, including seven women and two men aged 39 to 77, diagnosed with various cancers between 1999 and 2022 (see Table 1). The group included both trial-experienced and trial-naïve participants, as well as individuals currently affected by cancer and those in remission. Audio recordings were made using two devices for redundancy. Files were securely stored on encrypted university hard drives and deleted from recording devices. Only the research team had access to the data.

**Table 1:** Participant characteristics (N = 9) and cancer events (n = 11).

	N	(%)
Gender		
Female	7	(77.8)
Male	2	(22.2)
Age		
Between 39 and 49	1	(11.1)
Between 50 and 59	3	(33.3)
Between 60 and 69	4	(44.4)
70 or older	1	(11.1)
Cancer type^a^		
Breast	5	(45.5)
Hepatocellular	1	(9.1)
Gastrointestinal	1	(9.1)
Lung	1	(9.1)
Prostate	1	(9.1)
Thyroid	1	(9.1)
Melanoma	1	(9.1)
Diagnosed^a^		
Between 1999 and 2010	3	(27.3)
Between 2011 and 2015	2	(18.2)
Between 2016 and 2020	2	(18.2)
After 2020	4	(36.4)
Current cancer status		
Not affected	5	(55.6)
Affected	4	(44.4)
Trial experience		
Never been invited	4	(44.4)
Invited but declined	3	(33.3)
Participated but discontinued	1	(11.1)
Participated and completed	1	(11.1)

a Some study participants had more than one primary cancer; cancer-type and diagnosis-year counts are cancer events (n = 11) rather than unique participants. Percentages for gender, age, current status, and trial experience use the participant denominator (N = 9); percentages for cancer-type and diagnosis-year use the cancer-event denominator (n = 11).

Each session followed a four-stage structure based on Liamputtong [24]: pre-discussion, introduction, questioning, and debriefing. The setting was arranged to promote comfort and group cohesion, with participants addressed by first names and offered refreshments. The interview guide was developed based on existing literature and refined through patient and public involvement (PPI), including consultations with members of the Swiss Cancer Institute (SCI)'s patient advisory board. It contained open-ended questions addressing participants' attitudes, perceived barriers, motivations, expectations, and understanding of key trial concepts such as randomization, blinding, and placebo use (see Fig. S3 in the supplementary file). Sessions lasted approximately 90 min, and participants were encouraged to speak freely.

#### 2.2.3 Ethics and compensation

The study did not fall under the scope of the Swiss Human Research Act and received, therefore, a waiver from the Ethics Committee Northwest and Central Switzerland (EKNZ). Written informed consent was obtained from all participants. Confidentiality and data protection protocols were strictly followed. Each participant received a CHF 50 book voucher and refreshments.

#### 2.2.4 Data analysis

Audio recordings were transcribed verbatim and analysed using applied thematic analysis following Guest and colleagues [23]. The study team manually transcribed and coded the data independently. Initial codes were generated inductively and reviewed twice for consistency. Themes were refined through team discussions to ensure coherence and representativeness. NVivo software was used to manage and organise the data. A coding tree was developed, and illustrative quotations were selected to support key themes. Findings were reviewed for clarity and consistency in line with COREQ criteria [22]. Data saturation was not discussed due to recruitment challenges.

#### 2.2.5 Reliability and validity

To reduce bias, participants were reassured about confidentiality to encourage openness. Although external reviewers were not involved, objectivity was supported through a rigorous inductive approach and continuous cross-referencing of themes with the data. Reflexivity was practised throughout, with the research team reflecting on personal assumptions and their influence on interpretation. Recruitment and sampling limitations were transparently reported to support the study's credibility.

## 3 Results

Thematic analysis of the focus group data revealed two overarching themes: Perceived Barriers and Motivation, Expectations, and Understanding. Each theme comprised several sub-themes that reflect the complex factors influencing patients’ decisions about clinical trial participation (see Table 2). Direct quotations from participants illustrate the emotional, ethical, and practical dimensions of these experiences.

**Table 2:** Overarching themes and subthemes.

Overarching Theme	Subtheme	Description (based on participant accounts)
Barriers	Restricted access and concerns of financial incentives	Distrust in institutions and physicians, concerns about financial incentives, and lack of physician engagement in trial recruitment. Strict inclusion criteria excluded willing participants, causing frustration and disappointment.
	Informed and supported decision-making	Lack of clear communication, limited time to reflect, and absence of independent advice hindered informed consent.
	Treatment burden and resource constraints	Physical side effects, emotional exhaustion, frequent appointments, and scheduling conflicts made participation difficult.
	Family and emotional considerations	Participants feared burdening their families, adding emotional stress and complicating trial decisions.
Facilitators	Sense of purpose and moral commitment	Participants felt a moral obligation to contribute to research and saw participation as a way to give back to society.
	Patient-centred care and responsiveness	Supportive interactions and proactive responses to individual needs fostered trust and engagement.
	Anticipated personal benefits	Hope for access to effective treatments, financial compensation, and transparency about study outcomes motivated participation.
	Knowledge and access to information	Structured education, personal networks, and online resources empowered participants to seek and understand trial opportunities.

### 3.1 Perceived barriers to trial participation

Participants identified several barriers that hindered their willingness or ability to take part in clinical cancer trials. These included systemic and relational distrust, restrictive eligibility criteria, limited access to clear and independent information, time pressure during recruitment, treatment burden and lack of personal resources, and family-related emotional considerations.

#### 3.1.1 Restricted access and concerns of financial incentives

A recurring theme was distrust in institutions and health professionals, particularly around physicians' motivations. Some expressed concern that financial incentives might compromise ethical recruitment, as illustrated by a participant: “If a doctor recruits patients during his working hours and earns something from it … one could get the feeling that they're just enrolling people to make money” Others questioned the depth of physician engagement due to not being proposed any trial participation, as stated by another respondent: “So far, I have never been asked to participate … a surgeon just sees the surface … they stitch you up and say, ‘See you in a year.’”

Strict inclusion criteria were another source of frustration. Some participants reported being excluded from trials despite having relevant medical histories. One participant recounted: “I had signed up … but because only three lymph nodes were removed, which was too few for the study, I was excluded.”

#### 3.1.2 Informed and supported decision-making

Respondents considered clear and honest communication essential during recruitment. Participants wanted physicians to explain procedures, risks, and goals thoroughly. One participant urged: “Be honest, authentic, and put everything on the table … so that one can confidently say, ‘Yes, I agree.’ Reflection was also important to participants, who wanted physicians to consider the impact of their recommendations. One participant challenged the motivations behind recruitment by asking: “If this were your mother or your wife, would you do it?” Study participants also stressed the importance of receiving independent advice, separate from the study team. As one noted: “If it's something significant … I would want independent information – not just from the study team.” Others called for access to unbiased experts who could “place it into a broader context,” especially when patients lacked the energy or resources to research on their own.

Participants expressed frustration with rapid recruitment timelines. One participant shared: “I would have liked to say, ‘Thank you very much for the information, I'll take it home … ’ but instead, it was like, ‘I'll call you in 2 h’” The lack of time to reflect and consult with family and others was seen as a barrier. Another noted missed opportunities: “Between my surgery and the start of chemotherapy, there were four to six weeks … but I only found out about a trial two days before starting my treatment.”

#### 3.1.3 Treatment burden and limited personal resources

Some participants also described experiencing side effects as reasons for withdrawing from trials. One shared experiences of severe physical reactions, such as: “a brutal rash on my leg … everything was inflamed.” Others spoke about side effects affecting their sexuality, which carried a deeper psychological burden. As one participant shared: “I had my struggles with impotence … that was something that really hit me hard,” illustrating how trial participation can affect intimate aspects of life and self-esteem. For some, the practical demands of trial-related treatments were also a deterrent. Frequent injections and handling medical supplies were described as both exhaustive and intrusive: “Handling all those tiny needles and ampoules is difficult … you don't always have the patience for it.”

Already, the demands of routine cancer treatment, including frequent appointments and scheduling conflicts, were seen as major obstacles, not leaving much time for additional obligations such as trial participation. One participant described attending 21 appointments in a single month and choosing a study solely because it aligned with an existing visit: “That way, the procedures were done together, and I didn't have to schedule additional blood draws.” Another participant stated that study visits were incompatible with work responsibilities: “I work 90 percent, and if I had to invest too much time or risk feeling unwell, my answer would be a clear no.”

Chemotherapy was described as physically and emotionally exhausting. Participants reported difficulty with daily tasks and a loss of autonomy. Psychological responses shifted from initial shock to emotional fatigue. These cumulative strains result in perceptions that undertaking any additional responsibility, including clinical trial participation, would be unmanageable: “In the middle of therapy, I wouldn't have wanted to deal with anything more [clinical trial].”

Furthermore, family involvement introduced additional emotional complexity. Participants expressed a desire to protect their loved ones from added stress. One respondent shared: “You want to relieve your family, not put more burden on them.” For these individuals, trial participation was perceived as an added burden on their support network. The dual responsibility of managing their illness while continuing to care for loved ones intensified emotional stress and made the idea of joining a study feel overwhelming.

### 3.2 Facilitators of trial participation

#### 3.2.1 Feeling purpose and a moral obligation to participate

Perceptions of being a valid and contributing member of society were strong drivers of participation. Many viewed research as essential to medical progress and felt a moral obligation to contribute. One participant stated: “Without these studies, there is no progress,” while another reflected: “I clearly benefited from the fact that such studies exist.” Several described trial involvements as a way to give back: “Do good, and good comes back.” Others saw participation as a meaningful societal role: “This is my contribution to society – being able to do something good despite being ill.” Furthermore, trial involvement was seen not only as a personal contribution but also as a collaborative effort: 'We are working on something together … reaching something together – that has always been my positive approach to life.'

#### 3.2.2 Patient-centred care and responsiveness

Participants consistently emphasized the importance of patient-centred care and tangible benefits throughout trial involvement. Supportive and responsive interactions were highly valued, especially when healthcare professionals demonstrated attentiveness to individual needs. One participant described a particularly positive experience in which the study team identified an unmet nutritional need through a routine questionnaire: “They immediately noticed it through the questionnaire … boom, a nutritionist … ‘Let's get the doctor involved.’” This proactive response led to timely nutritional support and medical follow-up, reinforcing trust in the trial process and creating a sense of ‘being taken care of’. The sense that someone was actively monitoring her well-being and taking initiative was deeply reassuring: “It was like having another person look out for her.”

Other participants who were lacking this type of individualized care expressed a desire for basic support and follow-up. As one noted: “I would have found that beneficial … and I realize these considerations are very patient-centred.”

#### 3.2.3 Direct benefits from participation

Beyond contributing to research, participants hoped for personal benefits from trial participation. These included access to promising treatments, financial support, and transparency about study outcomes. One participant stated: “If I am in the placebo group and later it turns out that the study medication is effective, then I should also receive that medication.” Some participants expressed concern about receiving a placebo, fearing it might take away valuable treatment time: “If I only get a placebo, that's valuable time I've lost.” Nonetheless, participants expressed trust in the ethical safeguards of clinical research: “I don't believe they'd just throw us into a study and then toss us aside.”

Financial compensation for time or travel expenses related to the study was also discussed: “Especially for us retirees, it's important … at least covering expenses.” The CHF 50 voucher offered in this study was appreciated: “It's a small way of recognizing the effort … without having to ask for receipts.” Participants also emphasized the importance of non-financial benefits, particularly being informed about study outcomes. They expressed a desire for transparency and reciprocity, feeling that if they contributed to research, they should be kept informed and, where possible, benefiting from advances in medical knowledge. As one participant put it: “Patients should be informed … ‘If this study has a positive outcome, you will directly benefit from it.”

#### 3.2.4 Knowledge and access to information

Participants demonstrated varying levels of understanding of clinical trial concepts such as placebo, randomization, and blinding. While some had encountered these ideas through patient and public involvement courses in the past, they acknowledged that their familiarity had diminished over time: “I think people still kind of remember that … but honestly, I'm not super clear on all of it anymore.” There was a shared desire for broader education about clinical research: “These are things people really ought to know by now … just the basics – like what does ‘randomized’ mean? What's double-blind randomization?”

Participants described diverse experiences accessing information about clinical trials. Those who completed structured programs such as the EUPATI Patient Expert Course reported improved understanding: “The course definitely expanded my vocabulary.” Others relied on personal networks: “If I hadn't had my mom, who's a scientist, there are some words I still wouldn't understand.” Some described a basic understanding of trial procedures: “From a patient's perspective, I just knew they measured the baseline values, and then the study proceeded from there.”

In addition to passive learning, several participants actively sought out information using online registries to identify trials they could join. One remarked: “You have to check to find out what trials are currently running … ClinicalTrials.org,”, reflecting a proactive approach to participation. There was also support for preclinical research, including animal testing, which some viewed as a necessary step in the development of new treatments. As one participant put it: “I'm glad they do animal testing, even if it sucks for the animals too.”

## 4 Discussion

This study explored Swiss cancer patients' decision-making processes to clinical trial participation. Two main themes emerged: 1) Perceived barriers 2) and facilitators of trial participation. Barriers included systemic distrust of institutions and physicians, restrictive eligibility criteria, limited access to independent information, time pressure during recruitment, treatment burden, and family concerns. Facilitators were shaped by a sense of moral obligation and societal contribution, patient centred care and responsiveness from study teams, direct personal benefits such as access to promising treatments or financial support, and transparent communication. These findings highlight the tension between rigid trial structures and patients’ expectations for trust, reciprocity, and support, suggesting that greater responsiveness to patient perspectives is essential for improving recruitment and retention.

Our results are consistent with previous studies showing that patients often weigh their trust in physicians and the perceived motivations behind recruitment. Concerns about whether clinicians act in patients' best interests or are influenced by external incentives have been widely reported [6,12,25]. Mistrust is particularly pronounced among minority groups, where historical abuses and perceived lack of transparency have shaped skepticism toward clinical research [26]. Fears that financial incentives or institutional pressures could compromise ethical standards further reinforce doubts about trial invitations [27,28]. In addition, insufficient communication or perceived indifference from clinicians has been shown to erode trust and reduce willingness to participate [25,29]. Structural factors also play a role: Switzerland's decentralized healthcare system and lack of standardisation can hinder coordinated outreach and patient support [5]. Taken together, these reflections highlight how patients balance potential risks and benefits of participation, underscoring the importance of transparent communication, ethical reassurance, and meaningful engagement to foster trust and improve trial enrolment.

Treatment-related burdens and unmet support needs have been widely reported as reasons for trial withdrawal or non-adherence [30,31]. Proactive care, such as nutritional counselling and symptom monitoring, has been shown to foster safety and dignity, reinforcing patients’ confidence in the trial process [32,33]. Beyond physical symptoms, logistical challenges including work obligations, travel distance, and scheduling conflicts remain significant deterrents. Financial hardship, particularly out-of-pocket costs for travel, lodging, and medical expenses, is a leading cause of declining participation and early withdrawal [34]. In this context, financial recognition through travel reimbursement or flat-rate contributions was perceived as respectful and fair. Recruitment practices also require sensitivity: empathetic timing and flexibility during intensive treatment phases have been emphasized as critical to patient engagement [35].

Transparency emerged as another essential factor. Patients expected honest explanations and questioned whether physicians would recommend the same trials to their own families, echoing concerns described by Dellson and colleagues [13]. Physician involvement and encouragement were strong motivators, with patients more likely to enroll when their oncologist was the investigator or actively recommended the trial, underscoring the importance of a trust-based relationship [25,27,36]. Early access to information and independent advisory services were preferred to support informed decisions, consistent with Bell and Balneaves [37]. However, even highly educated individuals may struggle with registry platforms due to technical language and inconsistent formatting.

Family responsibilities added further emotional complexity, particularly among women who sought to protect loved ones from additional stress, consistent with Nielsen and Berthelsen [12]. Concerns about invasive procedures and unclear personal benefits also reduced willingness to participate [3]. Evidence suggests that flexible scheduling, clearer communication, and non-binding introductions can reduce anxiety and improve uptake [38,39]. Finally, a strong motivator across studies was the desire to contribute to society and medical progress, supporting future patients [12,40]. Yet, this altruistic motivation declined when communication and support were lacking, highlighting the need to treat participants as partners in research rather than passive subjects.

### 4.1 Implications

While motivation to participate in clinical trials is often strong, systemic and communicative shortcomings continue to hinder recruitment and retention. Addressing these barriers requires more inclusive communication strategies, flexible trial designs that accommodate patients’ daily lives, and accessible education to support informed decision-making.

This study illustrates the value of a patient-centred approach. Collaboration with the SCI's patient advisory board ensured that patient perspectives were integrated throughout the research process, while diverse sampling across cancer types captured a broad range of experiences. Including both trial-experienced and trial-naïve participants enriched the findings by highlighting differences in familiarity and expectations. Ethical procedures and fair compensation further supported trust, reinforcing the importance of transparency and reciprocity in clinical research.

### 4.2 Limitations and future directions

The study was limited to German-speaking regions, excluding perspectives from French- and Italian-speaking areas. Cultural and linguistic differences may influence attitudes toward clinical trial participation, and future studies should aim to include a broader regional and linguistic sample to capture more diverse experiences. Afternoon scheduling may have favoured retirees, reducing representation of younger and working participants. Recruitment challenges also resulted in a smaller sample than originally planned, which may limit the breadth of perspectives captured despite achieving thematic sufficiency. Focus groups encouraged open dialogue but may have constrained disclosure of sensitive experiences. Mixed-methods and longitudinal designs could capture individual trajectories and evolving attitudes more fully. Future research should also evaluate targeted interventions such as early information provision, independent advisory services, and caregiver support, which participants repeatedly identified as critical for informed decision-making and emotional readiness. Furthermore, future research should explore how trust in healthcare professionals and institutions affects trial participation across diverse populations. Given the emphasis participants placed on ethical reassurance and independent advice, studies could examine the impact of third-party counselling or decision aids. The emotional complexity of caregiving roles, especially among women, also warrants further investigation.

## 5 Conclusion

This study provides important insight into how Swiss cancer patients navigate decisions about clinical trial participation. Although a strong regulatory framework exists, gaps in communication, emotional support, and accessible information continue to shape willingness to engage. Improving participation requires transparent and empathetic communication, access to independent advisory services, and flexible trial designs that reflect individual circumstances. Education for both patients and healthcare professionals is critical to fostering a more inclusive and patient-centred research culture. Integrating patient perspectives into research processes and medical education will help ensure that cancer trials remain ethical, relevant, and responsive to evolving patient needs.

## CRediT authorship contribution statement

**Sandro Tiziano Stoffel:** Writing – review & editing, Writing – original draft, Validation, Supervision, Project administration, Methodology, Investigation, Formal analysis, Conceptualization. **Helene Seaward:** Writing – review & editing, Writing – original draft, Supervision, Methodology, Investigation, Formal analysis. **Aksha Balakrishnan:** Writing – review & editing, Writing – original draft, Validation, Software, Methodology, Investigation, Funding acquisition, Formal analysis, Data curation, Conceptualization.

## Funding

Department of Public Health of the University of Basel covered the ethics committee fees. The Swiss Cancer Institute (SCI) provided vouchers for participants and covered food and drink costs during the focus group interviews.

## Declaration of competing interest

The authors declare the following financial interests/personal relationships which may be considered as potential competing interests: Aksha Balakrishnan reports financial support for the ethics application was provided by Department of Public Health of the University of Basel. Aksha Balakrishnan reports financial support for the recruitment of the study participants and organization of the focus groups was provided by Swiss Cancer Institute (SCI). Other authors declare that they have no known competing financial interests or personal relationships that could have appeared to influence the work reported in this paper.

## Data Availability

The data that has been used is confidential.
