# Supplementary figures and images for: Understanding recruitment challenges in Swiss oncology trials: Patient voices from focus groups
Source: Contemp Clin Trials Commun. 2026 Jun 22;52:101661. doi: 10.1016/j.conctc.2026.101661 (PMC13316276; doi:10.1016/j.conctc.2026.101661)

Supplementary file

Figure S1. Invitation leaflet


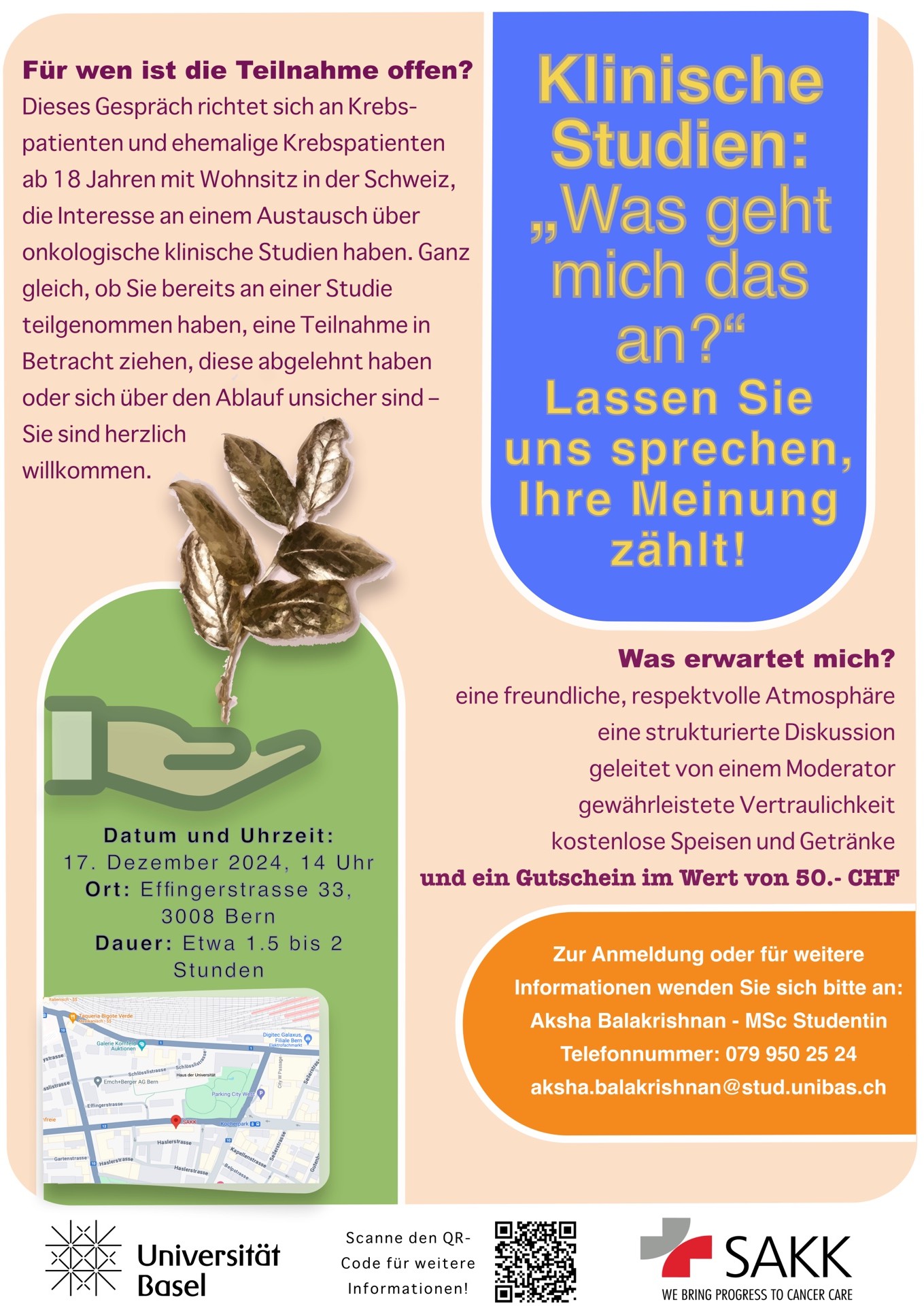


Figure S2. Invitation email


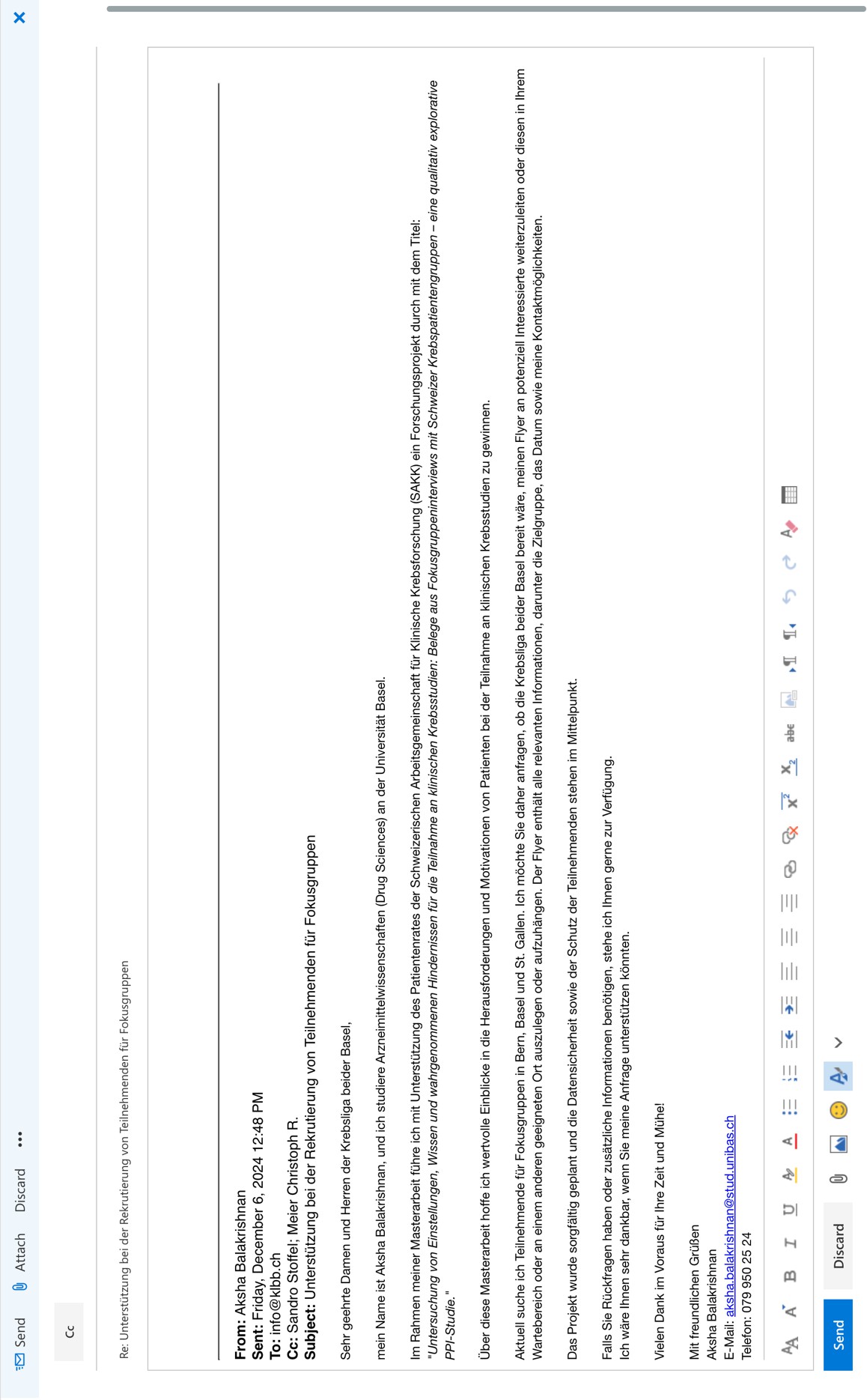


Figure S3. Guide


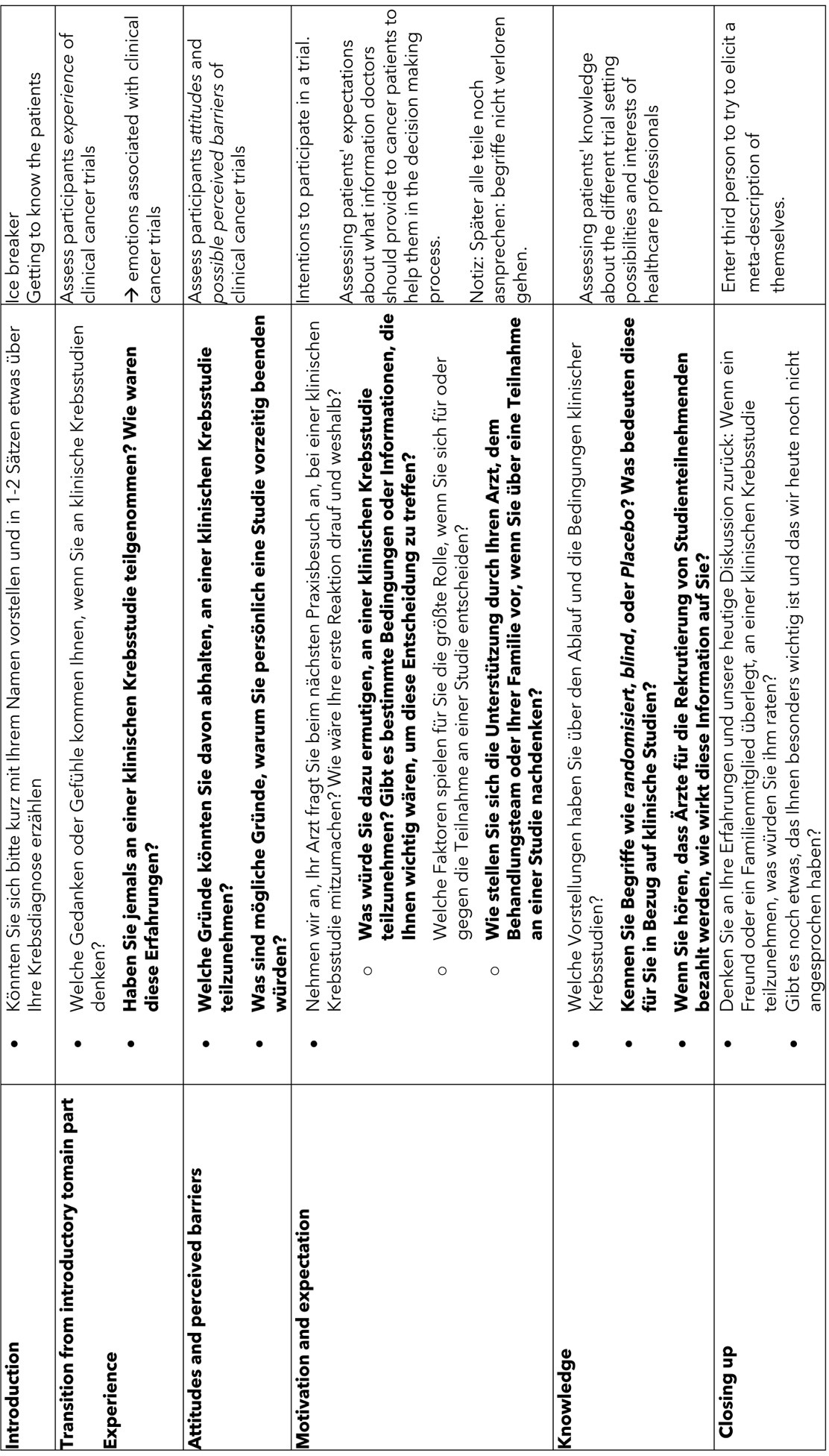

Supplement: Multimedia component 1 [file mmc1.docx]
